# A predictive model for depression in Chinese middle-aged and elderly people with arthritis

**DOI:** 10.1186/s12888-026-07864-x

**Published:** 2026-02-02

**Authors:** Li Yin, Kehong Pu, Wei Ke

**Affiliations:** Department of Emergency, The Second Affiliated Hospital of Chongqing, Medical University, Chongqing, 400000 China

**Keywords:** Depression, Arthritis, Middle-aged and elderly, Clinical prediction models, CHARLS

## Abstract

**Background:**

This study examines the prevalence of depression and its determinants among Chinese middle-aged and elderly arthritis patients, aiming to establish a theoretical foundation for enhancing their mental well-being and to inform the development of targeted prevention and intervention strategies.

**Methods:**

Data from the 2018 China Health and Retirement Longitudinal Study (CHARLS) were used for this study. We defined depression status in middle-aged and elderly arthritis patients as the dependent variable and included 16 predictor variables. The data were randomly divided into training and validation sets according to 7:3 ratio. LASSO and binary logistic regression analyses were performed on the training set to screen predictor variables and construct the model, which was then internally validated on the validation set.

**Results:**

This study included 1302 middle-aged and elderly arthritis patients. LASSO and binary logistic regression analysis were used to construct a prediction model for depression applicable to this population in China. The nomogram analysis revealed that female sex, middle age (45–59 years), poor self-rated health, being troubled by body pain, low life satisfaction, low marital satisfaction, low child satisfaction, and difficulties with instrumental activities of daily living (IADL) were risk factors for depression (*P* < 0.05). The area under the receiver operating characteristic curve(ROC) exceeded 0.70 in both the model training and internal validation phases, demonstrating the model’s high accuracy in predicting depression risk. In addition, decision curve analysis (DCA) and calibration curve analysis further confirmed the model’s practical value and validity.

**Conclusion:**

In this study, we identified that being female, middle-aged, having poor self-rated health, being troubled by body pain, dissatisfaction with life, marriage, and children, and difficulties with instrumental activities of daily living were risk factors for depression among middle-aged and elderly arthritis patients. We developed a predictive model based on these risk factors to facilitate early identification, intervention, and treatment for high-risk individuals.

**Supplementary Information:**

The online version contains supplementary material available at 10.1186/s12888-026-07864-x.

## Background

The challenge of aging is becoming increasingly prominent in the context of global economic development, and China is no exception [1]. Arthritis, a common disease among middle-aged and elderly people, increases in prevalence with age. International data show significant global variation in arthritis prevalence [2]. According to self-reported data from the World Health Organization’s Study on Global Ageing and Adult Health (SAGE), the prevalence in China is approximately 20% among people aged over 50 years [3, 4]. Studies have shown that depression is highly prevalent among middle-aged and older adults, with a prevalence exceeding 30% among people over 45 years of age in China [5, 6]. Depression is projected to be the leading cause of the global disease burden by 2023 [7].

Existing studies have revealed an association between arthritis and depression, indicating that arthritis patients have a higher risk of depression [8–12]. However, most studies have limitations regarding the inclusion of factors and risk quantification, making effective depression prevention difficult. In this study, we constructed a prediction model for depression in middle-aged and elderly Chinese arthritis patients using data from the China Health and Retirement Longitudinal Study (CHARLS), employing LASSO and binary logistic regression. The model identified predictors strongly associated with depression and visualized them using a nomogram, which facilitates patients’ self-assessment of risk and the adoption of interventions. Meanwhile, the model helps clinical staff quickly identify high-risk patients for the early diagnosis and intervention of depression.

## Method

### Data sources and model design

The China Health and Retirement Longitudinal Study (CHARLS) is a highly authoritative, large-scale interdisciplinary survey project in China.It is hosted by the National School of Development at Peking University and jointly implemented by the China Social Science Survey Center of Peking University and the Communist Youth League Committee of Peking University. Using a questionnaire survey as its core data collection method, the project covers 28 provinces and 150 districts/counties across China, focusing precisely on the middle-aged and elderly population aged 45 and above. Its content comprehensively includes multi-dimensional indicators such as personal basic information, family structure and economic support, health status, physical measurements, medical service utilization and medical insurance, work, retirement and pensions, income, consumption and assets, as well as basic community conditions. These data can be used to analyze China’s population aging issue, promote interdisciplinary research on aging, and provide a more scientific basis for the formulating and improving of relevant policies in China [13].

**Fig. 1:**
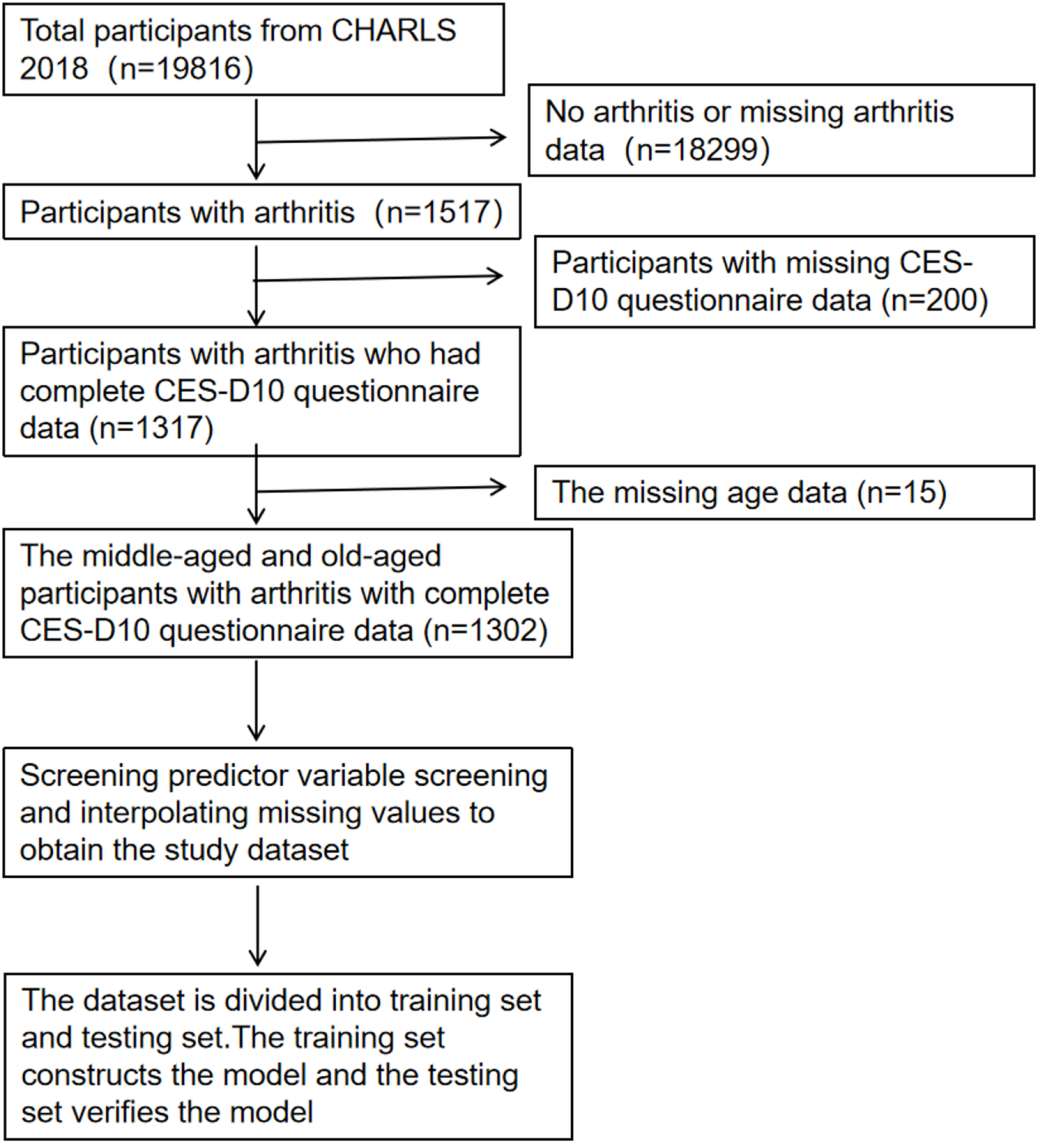
Flowchart of participant selection from the CHARLS. Note: CHARLS The China Health and Retirement Longitudinal Stud. CES-D10 Centre for Epidemiological Studies Depression Scale

For this study, we selected data from 2018 wave, from which we extracted information on the Centre for Epidemiological Studies Depression Scale (CES-D10), health behaviors, demographic factors, physical functioning, and social interactions among middle-aged and elderly individuals with arthritis. The inclusion criteria were as follows: (1) age ≥ 45 years; (2) a “yes"response to the questionnaire item “da007_13_: “Has a doctor ever told you that you have arthritis?“; and (3) a clear response to the depression scale (CES-D). Exclusion criteria were: (1) lack of information on arthritis, (2) lack of information on depression, and (3) lack of information on relevant covariates. A total of 1302 middle-aged and elderly patients with arthritis were included. The study was approved by the Biomedical Ethics Committee of Peking University (IRB00001052-11015), and informed consent was obtained from all respondents. The data screening process is shown in the flow chart in Fig. 1.

### Depression assessment

The CHARLS used a simplified 10-item version of the Centre for Epidemiological Studies Depression Scale (CES-D10) developed by RADLOFF [14], which has demonstrated high reliability and validity in previous studies and can be widely applicable to middle-aged and older populations [15]. The CES-D10 consists of 10 items, each scored from 0 (rarely or none of the time) to 3 (most or all of the time). The total score ranges from 0 to 30, with lower scores indicating fewer depressive symptoms. Studies have shown that a threshold score of 10 is reasonably sensitive and specific for Chinese older adults [16, 17]. Therefore, we defined a CES-D10 score of ≥ 10 as indicative of depression [18].

### Definition arthritis

Arthritis was defined based on self-reported physician diagnosis. All participants were asked, “Has a doctor ever told you that you have arthritis?” An affirmative answer was considered indicative of doctor-diagnosed arthritis.

### Measurement of covariates

The CHARLS 2018 collected data on socio-demographic characteristics (age, sex, marital status, education level, and place of residence), health-related lifestyles and behaviors (smoking, alcohol consumption, self-rated health status), hearing impairment, visual impairment, speech impairment, physical disability, being bothered by physical pain, self-reported physician-diagnosed comorbidities (hypertension, diabetes, dyslipidemia, chronic lung disease, liver disease, stroke, cancer, chronic kidney disease, digestive disease, and asthma), instrumental activities of daily living (IADL), childhood health status, marital satisfaction, life satisfaction, health satisfaction, air quality satisfaction, and child satisfaction.

### Incorporation of predictor variables and data set creation

Based on previous studies and the principle of maintaining missing values below 30% for predictor variables, we selected the following covariates. For demographic factors: gender, education level, marital status, religious affiliation, childhood health status, and residential address. For health behaviors: smoking, alcohol consumption, and self-rated health status. For physical functioning: vision, hearing, speech, physical disability, being bothered by physical pain, and IADL. For socialization: marital satisfaction, life satisfaction, health satisfaction, air quality satisfaction, and children satisfaction. Existing research suggests these variables may be associated with depression [19–24]. After compiling the predictor variables, multiple imputation was used to handle missing data.

### Logistic regression analysis and nomogram model development

A total of 1302 patients were included and randomly divided into training and validation groups in a 7:3 ratio. No significant differences in demographic and clinical characteristics were observed between the groups. LASSO regression analysis was performed on the training set to effectively screen predictors associated with depression while addressing multicollinearity. Subsequently, binary logistic regression analysis was used to identify key variables affecting depression. Based on the training set data, a nomogram model was constructed using the “rms” package in R, and its validity was tested on the validation set.

### Model evaluation

Discrimination and calibration were used to assess the predictive accuracy of the model. Harrell’s consistency index (C-index) and the Receiver Operating Characteristic Curve were generated to estimate the discrimination of the nomogram. A C-index close to 1 indicates high predictive power. The area under the ROC curve (AUC) was used to assess predictive efficacy. Calibration was evaluated using the Hosmer-Lemeshow test to determine the agreement between predicted and observed probabilities of depression [25]. Finally, the clinical utility of the nomogram was assessed via decision curve analysis (DCA) using bootstrap resampling (1000 replicates) [26].

### Statistical analysis

Independent t-tests were used to compare continuous data between the two groups. The unequal variance t-test (Welch’s t-test) was used for groups with unequal variances. For skewed distributions, the Mann-Whitny U test was used. Statistical analyses were performed using R Software v.4.4.0 (The R Project for Statistical Computing, http://www.r-project.org). Logistic regression analyses and nomogram construction were performed using the “rms” package. Two-sided P values < 0.05 were considered statistically significant.

## Results

### General characteristics of the data set

A total of 1302 middle-aged and older individuals with arthritis were included: 656 (50.4%) were not depressed and 646 (49.6%) had depression. Among those with depression, 239 (37.0%) were male and 407 (63.0%) were female; 303 (46.9%) were aged 45–59 years, 266 (41.2%) were 60–74 years, and 77 (11.9%) were ≥ 75 years. Baseline characteristics are shown in Table 1.

**Table 1:** Baseline characteristics of the study population

Predictor variable	No Depression	Depression
	*N* = 656	*N* = 646
Sex:		
Female	321 (48.9%)	407 (63.0%)
Male	335 (51.1%)	239 (37.0%)
Age:		
45–59	282 (43.0%)	303 (46.9%)
60–74	298 (45.4%)	266 (41.2%)
≥75	76 (11.6%)	77 (11.9%)
Smoking:		
No	552 (84.1%)	570 (88.2%)
Yes	104 (15.9%)	76 (11.8%)
Drinking:		
No	410 (62.5%)	442 (68.4%)
Yes	246 (37.5%)	204 (31.6%)
Residence Location:		
City	189 (28.8%)	145 (22.4%)
Rural	467 (71.2%)	501 (77.6%)
Education level:		
High school and higher	88 (13.4%)	60 (9.29%)
Illiterate	107 (16.3%)	171 (26.5%)
Junior high school and lower	461 (70.3%)	415 (64.2%)
Marital Status:		
Cohabitation	536 (81.7%)	472 (73.1%)
Living alone	120 (18.3%)	174 (26.9%)
Religious beliefs:		
No	587 (89.5%)	571 (88.4%)
Yes	69 (10.5%)	75 (11.6%)
Self-rated health:		
General	380 (57.9%)	286 (44.3%)
Good	128 (19.5%)	53 (8.20%)
Not good	148 (22.6%)	307 (47.5%)
Vision Problem:		
No	630 (96.0%)	593 (91.8%)
Yes	26 (3.96%)	53 (8.20%)
Hearing Problem:		
No	619 (94.4%)	589 (91.2%)
Yes	37 (5.64%)	57 (8.82%)
Speech Impediment:		
No	652 (99.4%)	632 (97.8%)
Yes	4 (0.61%)	14 (2.17%)
Disability:		
No	635 (96.8%)	605 (93.7%)
Yes	21 (3.20%)	41 (6.35%)
Chronic Comorbidities:		
1 kind	154 (23.5%)	184 (28.5%)
2 kinds and above	120 (18.3%)	141 (21.8%)
No	382 (58.2%)	321 (49.7%)
Life satisfaction:		
Dissatisfaction	18 (2.74%)	175 (27.1%)
Satisfaction	638 (97.3%)	471 (72.9%)
Health satisfaction:		
Dissatisfaction	156 (23.8%)	320 (49.5%)
Satisfaction	500 (76.2%)	326 (50.5%)
Marriage satisfaction:		
Dissatisfaction	72 (11.0%)	171 (26.5%)
Satisfaction	584 (89.0%)	475 (73.5%)
Chidren satisfaction:		
Dissatisfaction	13 (1.98%)	55 (8.51%)
Satisfaction	643 (98.0%)	591 (91.5%)
Air quality satisfaction:		
Dissatisfaction	101 (15.4%)	141 (21.8%)
Satisfaction	555 (84.6%)	505 (78.2%)
IADL:		
Difficulties	91 (13.9%)	192 (29.7%)
No Difficulties	565 (86.1%)	454 (70.3%)
Health during childhood:		
Good	490 (74.7%)	468 (72.4%)
Not good	166 (25.3%)	178 (27.6%)
Troubled with body pain:		
No	198 (30.2%)	105 (16.3%)
Yes	458 (69.8%)	541 (83.7%)

Note IADL: instrumental activities of daily living

### Subgroup analysis by age group (middle-aged vs. elderly)

We conducted additional subgroup analyses by age (middle-aged: 45–59 years; elderly: ≥60 years). The results revealed distinct age-related differences in risk factors for depression. Among the elderly group, depression risk was more strongly influenced by difficulties in instrumental activities of daily living (IADL), lower children satisfaction, and lower life satisfaction, highlighting the prominent role of functional limitations and socio-emotional factors in the mental health in older adults. In contrast, depression in the middle-aged group was more associated with work-family stress and living alone. Detailed data are provided in Supplementary Tables S1 (middle-aged), S2 (elderly) and S3(combined).

### Identification of predictors

**Fig. 2:**
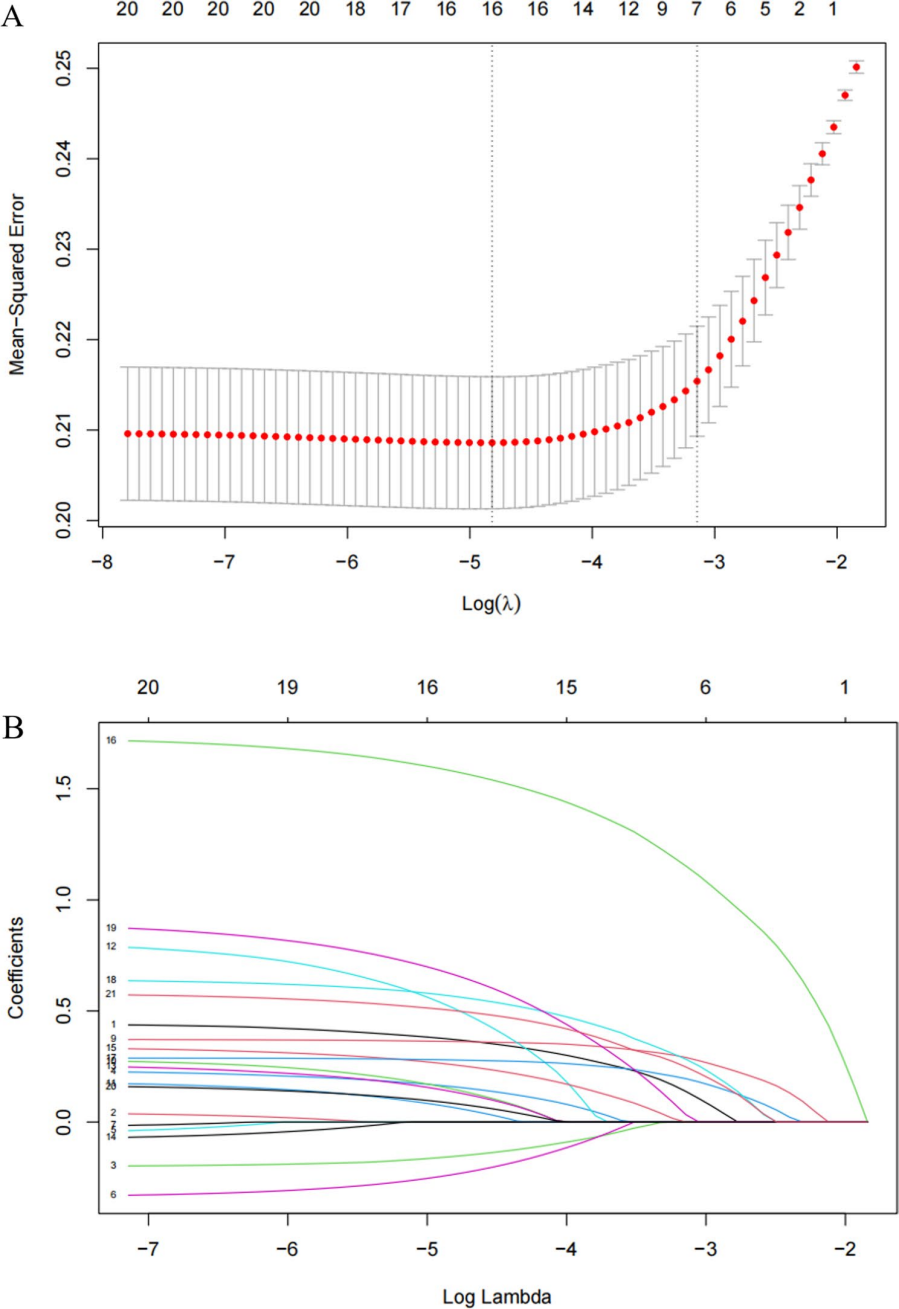
LASSO regression analysis combined with binary logistic regression for screening predictor variables. Note: (**A**) LASSO selection path plot. The left vertical dashed line indicates Log(λ) corresponding to the minimum error (lambda.1se); the right vertical dashed line indicates Log(λ) one standard error from the minimum error (lambda.min). Binomial Deviance represents the model’s binomial distribution loss function computed during cross-validation. (**B**) LASSO coefficient path plot shows the curve of regression coefficients versus Log(λ) as coefficients shrink. L1 Norm is the sum of absolute values of the model coefficients

Given the large number of variables, a two-step approach was used to filter clinical features. First, LASSO regression was performed for initial screening to identify potential predictors, avoiding overfitting and enhancing model robustness (Figs. 2A and B). This identified 16 candidate predictors: age, sex, education level, residence location, marital status, self-rated health, vision problem, drinking, speech impediment, trouble with body pain, IADL, marriage satisfaction, life satisfaction, health satisfaction, air quality satisfaction, and children satisfaction. Subsequently, binary logistic regression was performed on these 16 variables. Using a significance threshold of *P* < 0.05, eight variables were selected for the final model: sex, age, self-rated health, IADL, trouble with body pain, life satisfaction, marriage satisfaction, and children satisfaction.

### Construction and validation of a depression prediction model

**Fig. 3:**
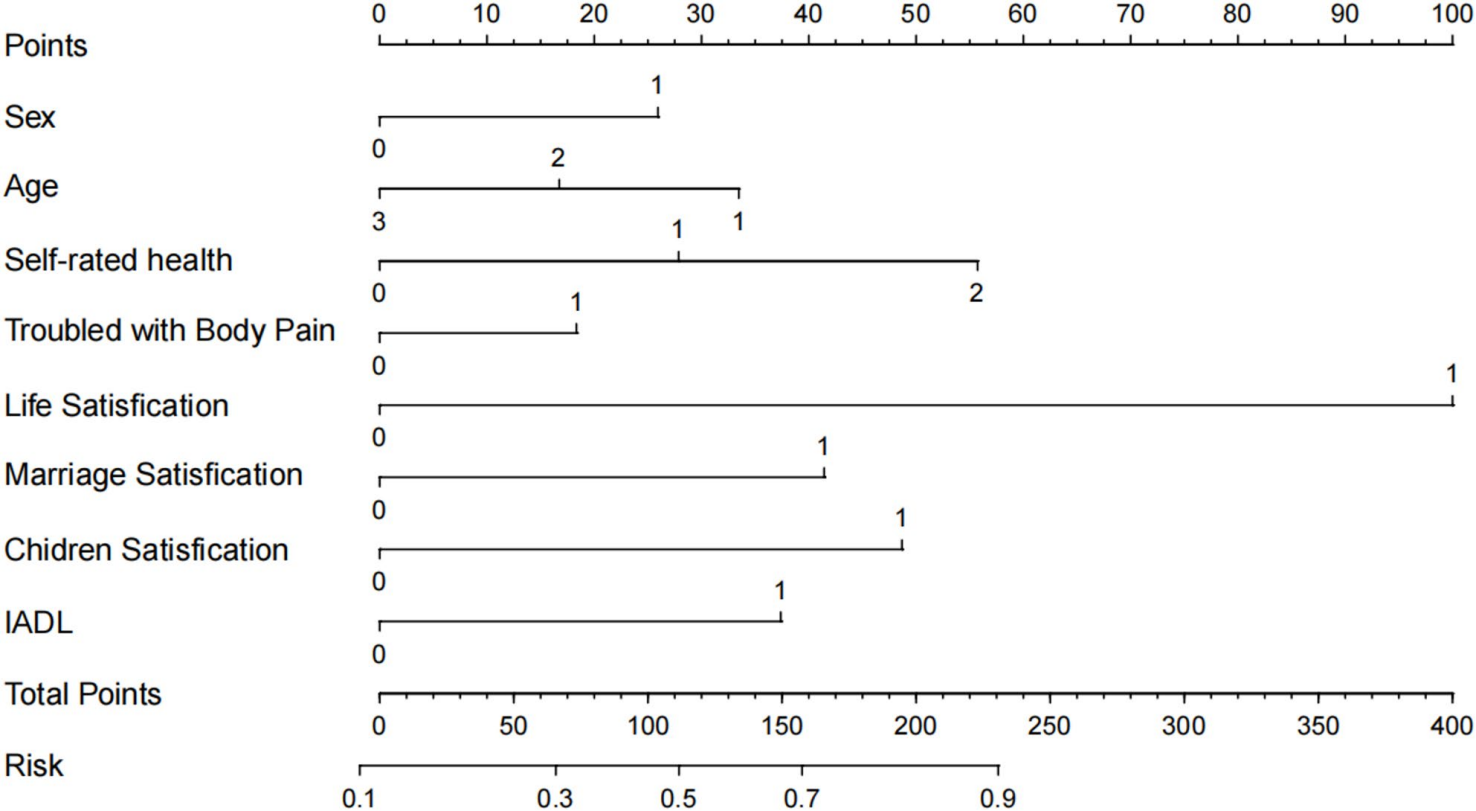
Nomogram for predicting the risk of depression among middle-aged and elderly patients with arthritis. Note: The total points from each predictor estimate the probability of depression. IADL: instrumental activities of daily living

After determining the final variables, we constructed the model in R using the “rms” package and generated a nomogram (Fig. 3).

### Predictive model validation discrimination

**Fig. 4:**
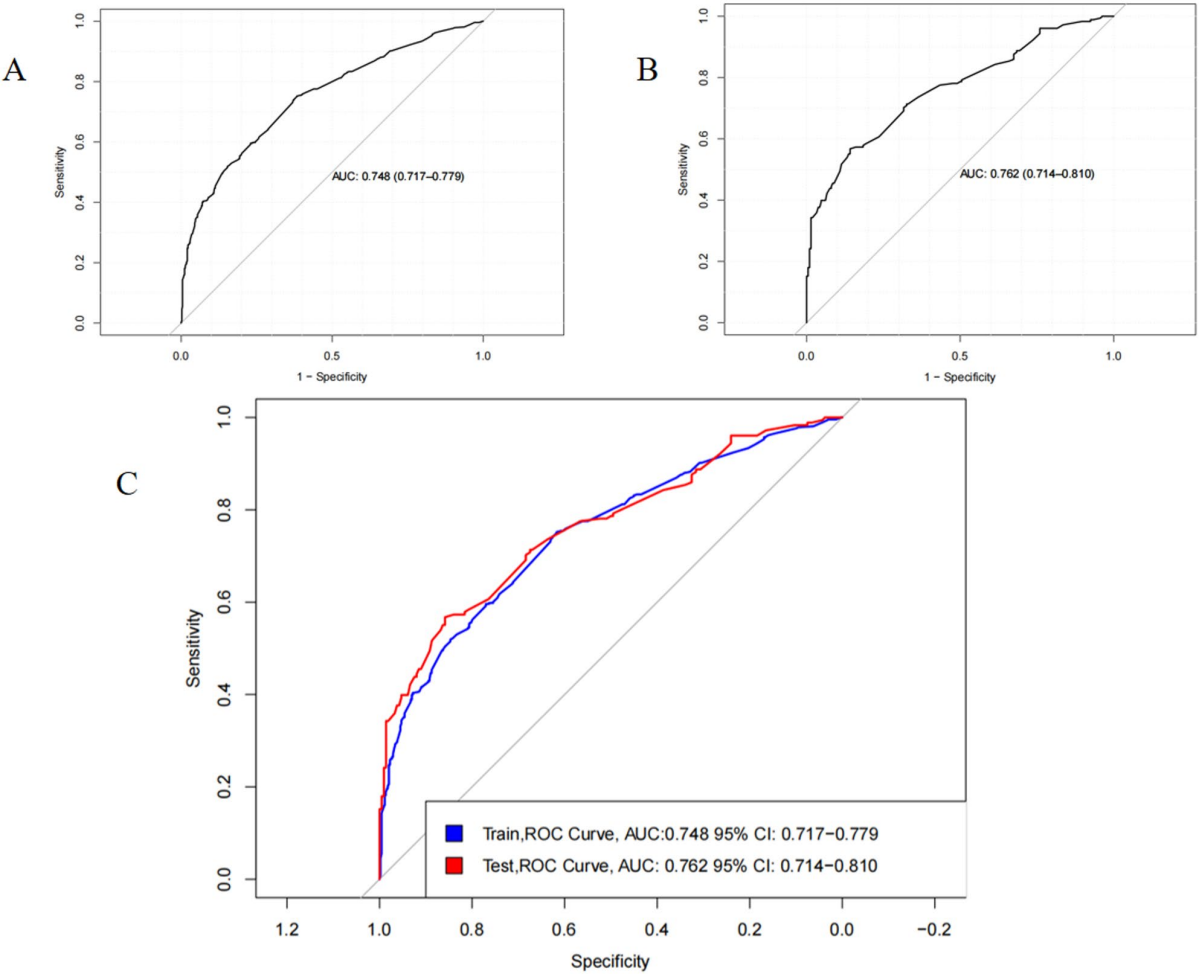
The area under the receiver operating characteristic curve [AUC] for the discrimination of the model. Note: [**A**] The training set, 0.748 [95% CI = 0.717 − 0.779]. [**B**] The validation set, 0.762 [95% CI = 0.714 − 0.810]

For the samples, the C-index was 0.748 (95% CI: 0.717–0.779) in the training set and 0.762 (95% CI: 0.714–0.810) in the validation set, indicating good discriminatory power. The AUC values were determined to assess the discriminatory nature of the column-line plots. In the training set, the AUC of the column-line diagram for predicting depression was: 0.748 (95% CI: 0.717–0.779). In the validation set, the AUC remained high at 0.762 (95% CI: 0.714–0.810), further supporting the strong discriminative power of the model (Fig. 4).

### Calibration and clinical use of the prediction model

**Fig. 5:**
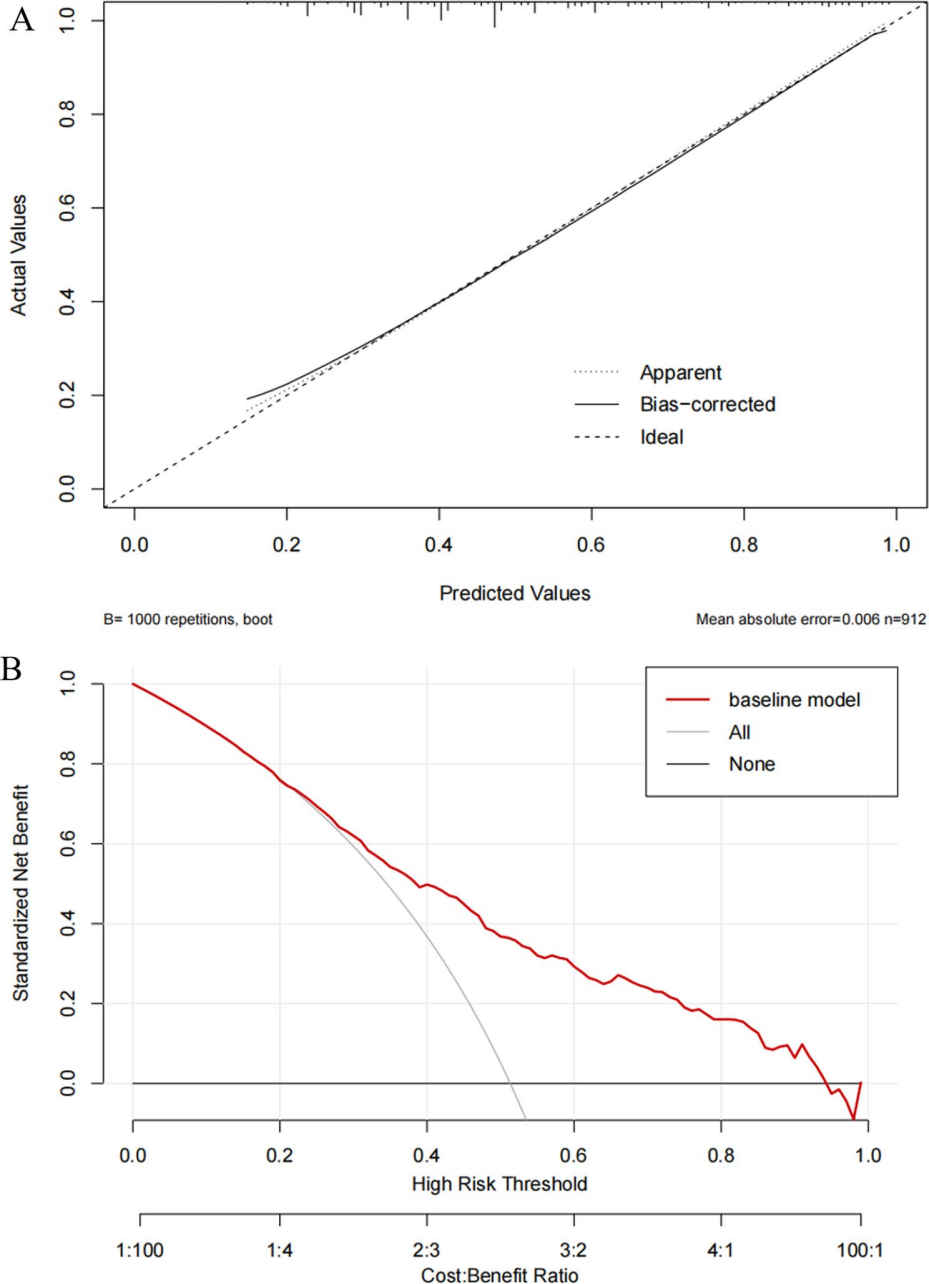
Calibration curves and decision curve analysis (DCA) of the depression risk prediction model. Note (**A**) Calibration curve: The x-axis represents the predicted probability of depression; the y-axis represents the actual observed probability. The curve assesses the agreement between predicted and observed probabilities. (**B**) DCA: The x-axis represents the high-risk threshold; the y-axis represents the standardized net benefit. The plot compares the net benefit of the model against treat-all or treat-none strategies across different thresholds

The mean absolute error of the model mean calibration curve was 0.006, as shown in Fig. 5A.The DCA curve showed that the model constructed using the eight predictor variables had higher net benefit across a wide range of thresholds compared to using a single predictor, indicating greater clinical utility (Fig. 5B).

## Discussion

CHARLS is one of the most representative socio-demographic surveys in China, known for its long duration, wide scope, and diverse variables. In this study, the CHARLS 2018 data revealed a strong association between arthritis and depression in the middle-aged and elderly population. We found that the prevalence of depression among arthritis patients was as high as 49.6% among Chinese middle-aged and older adults aged 45 years and older, with more than 77% of depressed patients living in rural areas where healthcare resources are relatively scarce. Age is a key factor influencing depression, with the highest prevalence observed in those aged 45–59 years, possibly related to higher life and work pressures in this group [27]. Furthermore, women had a significantly higher risk of depression than men in middle and old age, which may be associated with longer life expectancy, higher rates of widowhood, and physiological changes such as perimenopausal syndrome. Therefore, greater attention should be paid to the mental health of middle-aged and elderly women, with calls for enhanced disease prevention and social support.

Our subgroup analysis further revealed differences in risk factors across age groups. Depression risk among elderly arthritis patients (≥ 60 years) was more strongly influenced by functional disability (IADL) and social support factors (e.g., child satisfaction), likely related to physiological decline and social role transitions common in this age group. In contrast, depression among middle-aged patients (45–59 years) was more associated with work-family stress and living alone. These findings highlight the importance of developing age-specific strategies in clinical practice and public health interventions.

Self-rated health status, an important indicator of perceived health recommended by the WHO for health surveys [28], was confirmed in our study as a factor in depression development among middle-aged and elderly arthritis patients. This aligns with findings by Huang et al. [29], indicating that older adults with poorer self-rated health face a higher depression risk. Therefore, healthcare and community workers should strengthen health status assessments in this population. Chronic physical pain is another risk factor. Chronic pain not only threatens physical health but may also affect psychological status and social functioning, including sleep qualit y, work capacity, social activities, and interpersonal relationships. These factors can lead to helplessness, hopelessness, reduced life satisfaction, and strained family and marital relationships.

Assessment of instrumental activities of daily living (IADL) is essential for understanding independent living ability in older adults. We found that declining IADL was associated with increased depression risk in middle-aged and elderly arthritis patients, which coincides with early markers of cognitive impairment [30]. Additionally, lack of social activities was associated with a higher incidence of depression in both age groups, suggesting that reduced social participation lowers quality of life and increases depression risk.

We found that arthritis patients with high life satisfaction had a lower risk of depression. Similarly, marital satisfaction was negatively associated with depression risk. Previous studies have demonstrated that arthritis significantly reduces quality of life in middle-aged and elderly patients, thereby increasing depression risk [30]. Min et al. [31] stated that spousal support and marital satisfaction are moderating factors in depression development among patients with chronic diseases.

The higher prevalence of arthritis in our study may be due to regional variations in diagnosis and self-reporting biases. CHARLS includes rural populations with limited healthcare access, potentially leading to under-diagnosis or over-reporting of symptoms.

Our study also has some limitations. Firstly, we did not consider possible changes in the variables over follow-up time when examining the relationship between each baseline characteristic and depression. Second, this study used secondary data from the China Health and Retirement Longitudinal Study (CHARLS), which may have limitations such as recall bias and unmeasured confounding variables. For instance, both arthritis and depression status were self-reported by the participants, and the results may deviate from clinical diagnoses. Third, due to data limitations, this study failed to account for the duration and severity of arthritis. To gain deeper insights into the arthritis-depression relationship, future research should integrate objective clinical measures, such as radiographic assessment of disease severity and quantification of pain intensity. Finally, our model was developed based on data from China, which makes it difficult to generalize this study to other countries.

### Future research directions

It is also important to note that our model building strategy prioritized clinical interpretability and utility, leading to the selection of logistic regression. Future research could beneficially expand on this work by incorporating a comparative analysis of other advanced machine learning algorithms (e.g., Random Forest, XGBoost) to further optimize predictive performance, provided that model interpretability is maintained.

## Conclusion

In this study, we identified predictive factors for depression in middle-aged and elderly patients with arthritis, including female sex, age 45–59 years, poor self-rated health, physical pain, dissatisfaction with life, marriage, and children, and difficulties with instrumental activities of daily living. Based on these factors, a predictive model was constructed to estimate the risk probability of depression. The model also suggests a potential pathway for self-intervention to reduce risk. Clinicians can use the provided nomogram to quickly identify high-risk patients, enabling early diagnosis and intervention and thereby improving treatment efficiency.

## Supplementary Information

Below is the link to the electronic supplementary material.

Supplementary Material 1

Supplementary Material 2

Supplementary Material 3

## Data Availability

The data for this article comes from the China Health and Retirement Longitudinal Study database for 2018. Available from https://charls.pku.edu.cn/en/.
