# Supplementary material for: A predictive model for depression in Chinese middle-aged and elderly people with arthritis
Source: BMC Psychiatry. 2026 Feb 2;26:221. doi: 10.1186/s12888-026-07864-x (PMC12955044; doi:10.1186/s12888-026-07864-x)
Supplement: Supplementary file 1 — Supplementary Material 1 [file 12888_2026_7864_MOESM1_ESM.docx]

Supplementary Table S1 (middle-aged) **Baseline characteristics of the study population**

| **Predictor Variable** | **No Depression** | **Depression** | **OR（95% CI）** | **p** |
| --- | --- | --- | --- | --- |
|  | ***N=282*** | ***N=303*** |  |  |
| Sex: |  |  |  | 0.002 |
| Female | 142 (50.4%) | 192 (63.4%) | Ref. |  |
| Male | 140 (49.6%) | 111 (36.6%) | 0.59 [0.42;0.82] |  |
| Smoking: |  |  |  | 0.735 |
| No | 243 (86.2%) | 265 (87.5%) | Ref. |  |
| Yes | 39 (13.8%) | 38 (12.5%) | 0.89 [0.55;1.45] |  |
| Drinking: |  |  |  | 0.407 |
| No | 174 (61.7%) | 198 (65.3%) | Ref. |  |
| Yes | 108 (38.3%) | 105 (34.7%) | 0.85 [0.61;1.20] |  |
| Residence Location: |  |  |  | 0.207 |
| City | 73 (25.9%) | 64 (21.1%) | Ref. |  |
| Rural | 209 (74.1%) | 239 (78.9%) | 1.30 [0.89;1.92] |  |
| Education level: |  |  |  | 0.008 |
| high school and higher | 42 (14.9%) | 32 (10.6%) | Ref. |  |
| illiterate | 22 (7.80%) | 47 (15.5%) | 2.78 [1.41;5.59] |  |
| junior high school and lower | 218 (77.3%) | 224 (73.9%) | 1.35 [0.82;2.23] |  |
| Marital status: |  |  |  | 0.035 |
| cohabitation | 239 (84.8%) | 235 (77.6%) | Ref. |  |
| living alone | 43 (15.2%) | 68 (22.4%) | 1.61 [1.06;2.46] |  |
| Religious beliefs: |  |  |  | 0.238 |
| No | 257 (91.1%) | 266 (87.8%) | Ref. |  |
| Yes | 25 (8.87%) | 37 (12.2%) | 1.43 [0.84;2.47] |  |
| Self-rated health: |  |  |  | <0.001 |
| general | 171 (60.6%) | 152 (50.2%) | Ref. |  |
| good | 53 (18.8%) | 23 (7.59%) | 0.49 [0.28;0.83] |  |
| not good | 58 (20.6%) | 128 (42.2%) | 2.48 [1.70;3.64] |  |
| Vision Problem: |  |  |  | 0.043 |
| No | 275 (97.5%) | 284 (93.7%) | Ref. |  |
| Yes | 7 (2.48%) | 19 (6.27%) | 2.59 [1.11;6.80] |  |
| Hearing Problem: |  |  |  | 0.326 |
| No | 268 (95.0%) | 281 (92.7%) | Ref. |  |
| Yes | 14 (4.96%) | 22 (7.26%) | 1.49 [0.75;3.06] |  |
| Speech Impediment: |  |  |  | 0.625 |
| No | 281 (99.6%) | 300 (99.0%) | Ref. |  |
| Yes | 1 (0.35%) | 3 (0.99%) | 2.58 [0.30;7.42] |  |
| Disability: |  |  |  | 0.045 |
| No | 278 (98.6%) | 289 (95.4%) | Ref. |  |
| Yes | 4 (1.42%) | 14 (4.62%) | 3.27 [1.14;12.0] |  |
| Chronic Comorbidities: |  |  |  | 0.086 |
| 1 kind | 72 (25.5%) | 77 (25.4%) | Ref. |  |
| 2 kinds and above | 34 (12.1%) | 56 (18.5%) | 1.54 [0.90;2.64] |  |
| no | 176 (62.4%) | 170 (56.1%) | 0.90 [0.61;1.33] |  |
| Life satisfaction: |  |  |  | <0.001 |
| dissatisfaction | 12 (4.26%) | 93 (30.7%) | Ref. |  |
| satisfaction | 270 (95.7%) | 210 (69.3%) | 0.10 [0.05;0.18] |  |
| Health satisfaction: |  |  |  | <0.001 |
| dissatisfaction | 75 (26.6%) | 157 (51.8%) | Ref. |  |
| satisfaction | 207 (73.4%) | 146 (48.2%) | 0.34 [0.24;0.48] |  |
| Marriage satisfaction: |  |  |  | 0.003 |
| dissatisfaction | 21 (7.45%) | 48 (15.8%) | Ref. |  |
| satisfaction | 261 (92.6%) | 255 (84.2%) | 0.43 [0.25;0.73] |  |
| Chidren satisfaction: |  |  |  | 0.014 |
| dissatisfaction | 7 (2.48%) | 22 (7.26%) | Ref. |  |
| satisfaction | 275 (97.5%) | 281 (92.7%) | 0.33 [0.13;0.75] |  |
| Air quality satisfaction: |  |  |  | 0.015 |
| dissatisfaction | 49 (17.4%) | 79 (26.1%) | Ref. |  |
| satisfaction | 233 (82.6%) | 224 (73.9%) | 0.60 [0.40;0.89] |  |
| IADL: |  |  |  | <0.001 |
| Difficulties | 20 (7.09%) | 53 (17.5%) | Ref. |  |
| No Difficulties | 262 (92.9%) | 250 (82.5%) | 0.36 [0.21;0.62] |  |
| Health during childhood: |  |  |  | 0.063 |
| good | 220 (78.0%) | 215 (71.0%) | Ref. |  |
| not good | 62 (22.0%) | 88 (29.0%) | 1.45 [1.00;2.12] |  |
| Troubled with body pain: |  |  |  | <0.001 |
| no | 79 (28.0%) | 42 (13.9%) | Ref. |  |
| yes | 203 (72.0%) | 261 (86.1%) | 2.41 [1.60;3.69] |  |
