# Supplementary material for: A predictive model for depression in Chinese middle-aged and elderly people with arthritis
Source: BMC Psychiatry. 2026 Feb 2;26:221. doi: 10.1186/s12888-026-07864-x (PMC12955044; doi:10.1186/s12888-026-07864-x)
Supplement: Supplementary file 2 — Supplementary Material 2 [file 12888_2026_7864_MOESM2_ESM.docx]

Supplementary Table S2 (elderly) **Baseline characteristics of the study population**

| **Predictor Variable** | **No Depression** | **Depression** | **OR （95% CI）** | **p** |
| --- | --- | --- | --- | --- |
|  | ***N=374*** | ***N=343*** |  |  |
| Sex: |  |  |  | <0.001 |
| Female | 179 (47.9%) | 215 (62.7%) | Ref. |  |
| Male | 195 (52.1%) | 128 (37.3%) | 0.55 [0.41;0.74] |  |
| Smoking: |  |  |  | 0.022 |
| No | 309 (82.6%) | 305 (88.9%) | Ref. |  |
| Yes | 65 (17.4%) | 38 (11.1%) | 0.59 [0.38;0.91] |  |
| Drinking: |  |  |  | 0.027 |
| No | 236 (63.1%) | 244 (71.1%) | Ref. |  |
| Yes | 138 (36.9%) | 99 (28.9%) | 0.69 [0.51;0.95] |  |
| Residence Location: |  |  |  | 0.033 |
| City | 116 (31.0%) | 81 (23.6%) | Ref. |  |
| Rural | 258 (69.0%) | 262 (76.4%) | 1.45 [1.04;2.03] |  |
| Education level: |  |  |  | <0.001 |
| high school and higher | 46 (12.3%) | 28 (8.16%) | Ref. |  |
| illiterate | 85 (22.7%) | 124 (36.2%) | 2.38 [1.39;4.16] |  |
| junior high school and lower | 243 (65.0%) | 191 (55.7%) | 1.29 [0.78;2.16] |  |
| Marital status: |  |  |  | 0.002 |
| cohabitation | 297 (79.4%) | 237 (69.1%) | Ref. |  |
| living alone | 77 (20.6%) | 106 (30.9%) | 1.72 [1.23;2.43] |  |
| Religious beliefs: |  |  |  | 0.864 |
| No | 330 (88.2%) | 305 (88.9%) | Ref. |  |
| Yes | 44 (11.8%) | 38 (11.1%) | 0.93 [0.59;1.48] |  |
| Self-rated health: |  |  |  | <0.001 |
| general | 209 (55.9%) | 134 (39.1%) | Ref. |  |
| good | 75 (20.1%) | 30 (8.75%) | 0.63 [0.38;1.00] |  |
| not good | 90 (24.1%) | 179 (52.2%) | 3.09 [2.22;4.33] |  |
| Vision Problem: |  |  |  | 0.020 |
| No | 355 (94.9%) | 309 (90.1%) | Ref. |  |
| Yes | 19 (5.08%) | 34 (9.91%) | 2.05 [1.15;3.74] |  |
| Hearing Problem: |  |  |  | 0.064 |
| No | 351 (93.9%) | 308 (89.8%) | Ref. |  |
| Yes | 23 (6.15%) | 35 (10.2%) | 1.73 [1.00;3.03] |  |
| Speech Impediment: |  |  |  | 0.040 |
| No | 371 (99.2%) | 332 (96.8%) | Ref. |  |
| Yes | 3 (0.80%) | 11 (3.21%) | 3.94 [1.20;18.4] |  |
| Disability: |  |  |  | 0.089 |
| No | 357 (95.5%) | 316 (92.1%) | Ref. |  |
| Yes | 17 (4.55%) | 27 (7.87%) | 1.79 [0.96;3.41] |  |
| Chronic Comorbidities: |  |  |  | 0.005 |
| 1 kind | 82 (21.9%) | 107 (31.2%) | Ref. |  |
| 2 kinds and above | 86 (23.0%) | 85 (24.8%) | 0.76 [0.50;1.15] |  |
| no | 206 (55.1%) | 151 (44.0%) | 0.56 [0.39;0.80] |  |
| Life satisfaction: |  |  |  | <0.001 |
| dissatisfaction | 6 (1.60%) | 82 (23.9%) | Ref. |  |
| satisfaction | 368 (98.4%) | 261 (76.1%) | 0.05 [0.02;0.11] |  |
| Health satisfaction: |  |  |  | <0.001 |
| dissatisfaction | 81 (21.7%) | 163 (47.5%) | Ref. |  |
| satisfaction | 293 (78.3%) | 180 (52.5%) | 0.31 [0.22;0.42] |  |
| Marriage satisfaction: |  |  |  | <0.001 |
| dissatisfaction | 51 (13.6%) | 123 (35.9%) | Ref. |  |
| satisfaction | 323 (86.4%) | 220 (64.1%) | 0.28 [0.19;0.41] |  |
| Chidren satisfaction: |  |  |  | <0.001 |
| dissatisfaction | 6 (1.60%) | 33 (9.62%) | Ref. |  |
| satisfaction | 368 (98.4%) | 310 (90.4%) | 0.16 [0.06;0.35] |  |
| Air quality satisfaction: |  |  |  | 0.154 |
| dissatisfaction | 52 (13.9%) | 62 (18.1%) | Ref. |  |
| satisfication | 322 (86.1%) | 281 (81.9%) | 0.73 [0.49;1.09] |  |
| IADL: |  |  |  | <0.001 |
| Difficulties | 71 (19.0%) | 139 (40.5%) | Ref. |  |
| No Difficulties | 303 (81.0%) | 204 (59.5%) | 0.34 [0.25;0.48] |  |
| Health during Childhood: |  |  |  | 0.698 |
| good | 270 (72.2%) | 253 (73.8%) | Ref. |  |
| not good | 104 (27.8%) | 90 (26.2%) | 0.92 [0.66;1.29] |  |
| Troubled with body pain: |  |  |  | <0.001 |
| no | 119 (31.8%) | 63 (18.4%) | Ref. |  |
| yes | 255 (68.2%) | 280 (81.6%) | 2.07 [1.46;2.95] |  |
