# Supplementary material for: A predictive model for depression in Chinese middle-aged and elderly people with arthritis
Source: BMC Psychiatry. 2026 Feb 2;26:221. doi: 10.1186/s12888-026-07864-x (PMC12955044; doi:10.1186/s12888-026-07864-x)
Supplement: Supplementary file 3 — Supplementary Material 3 [file 12888_2026_7864_MOESM3_ESM.docx]

Supplementary Table S3 (combined) **Baseline characteristics of the study population**

| **Predictor variable** | **No Depression** | **Depression** | **OR （95% CI）** | **p** |
| --- | --- | --- | --- | --- |
|  | ***N=656*** | ***N=646*** |  |  |
| Sex: |  |  |  | <0.001 |
| Female | 321 (48.9%) | 407 (63.0%) | Ref. |  |
| Male | 335 (51.1%) | 239 (37.0%) | 0.56 [0.45;0.70] |  |
| Age: |  |  |  | 0.287 |
| 45-59 | 282 (43.0%) | 303 (46.9%) | Ref. |  |
| 60-74 | 298 (45.4%) | 266 (41.2%) | 0.83 [0.66;1.05] |  |
| ≥75 | 76 (11.6%) | 77 (11.9%) | 0.94 [0.66;1.35] |  |
| Smoking: |  |  |  | 0.040 |
| No | 552 (84.1%) | 570 (88.2%) | Ref. |  |
| Yes | 104 (15.9%) | 76 (11.8%) | 0.71 [0.51;0.97] |  |
| Drinking: |  |  |  | 0.029 |
| No | 410 (62.5%) | 442 (68.4%) | Ref. |  |
| Yes | 246 (37.5%) | 204 (31.6%) | 0.77 [0.61;0.97] |  |
| Residence Location: |  |  |  | 0.010 |
| City | 189 (28.8%) | 145 (22.4%) | Ref. |  |
| Rural | 467 (71.2%) | 501 (77.6%) | 1.40 [1.09;1.80] |  |
| Education level: |  |  |  | <0.001 |
| high school and higher | 88 (13.4%) | 60 (9.29%) | Ref. |  |
| illiterate | 107 (16.3%) | 171 (26.5%) | 2.34 [1.56;3.53] |  |
| junior high school and lower | 461 (70.3%) | 415 (64.2%) | 1.32 [0.93;1.89] |  |
| Marital Status: |  |  |  | <0.001 |
| cohabitation | 536 (81.7%) | 472 (73.1%) | Ref. |  |
| living alone | 120 (18.3%) | 174 (26.9%) | 1.65 [1.27;2.15] |  |
| Religious beliefs: |  |  |  | 0.590 |
| No | 587 (89.5%) | 571 (88.4%) | Ref. |  |
| Yes | 69 (10.5%) | 75 (11.6%) | 1.12 [0.79;1.58] |  |
| Self_rated health: |  |  |  | <0.001 |
| general | 380 (57.9%) | 286 (44.3%) | Ref. |  |
| good | 128 (19.5%) | 53 (8.20%) | 0.55 [0.38;0.78] |  |
| not good | 148 (22.6%) | 307 (47.5%) | 2.75 [2.15;3.54] |  |
| Vision Problem: |  |  |  | 0.002 |
| No | 630 (96.0%) | 593 (91.8%) | Ref. |  |
| Yes | 26 (3.96%) | 53 (8.20%) | 2.16 [1.34;3.55] |  |
| Hearing Problem: |  |  |  | 0.035 |
| No | 619 (94.4%) | 589 (91.2%) | Ref. |  |
| Yes | 37 (5.64%) | 57 (8.82%) | 1.62 [1.06;2.50] |  |
| Speech Impediment: |  |  |  | 0.030 |
| No | 652 (99.4%) | 632 (97.8%) | Ref. |  |
| Yes | 4 (0.61%) | 14 (2.17%) | 3.51 [1.24;12.8] |  |
| Disability: |  |  |  | 0.011 |
| No | 635 (96.8%) | 605 (93.7%) | Ref. |  |
| Yes | 21 (3.20%) | 41 (6.35%) | 2.04 [1.20;3.56] |  |
| Chronic Comorbidities: |  |  |  | 0.008 |
| 1 kind | 154 (23.5%) | 184 (28.5%) | Ref. |  |
| 2 kinds and above | 120 (18.3%) | 141 (21.8%) | 0.98 [0.71;1.36] |  |
| no | 382 (58.2%) | 321 (49.7%) | 0.70 [0.54;0.91] |  |
| Life Satisfaction: |  |  |  | <0.001 |
| dissatisfaction | 18 (2.74%) | 175 (27.1%) | Ref. |  |
| satisfaction | 638 (97.3%) | 471 (72.9%) | 0.08 [0.04;0.12] |  |
| Health Satisfaction: |  |  |  | <0.001 |
| dissatisfaction | 156 (23.8%) | 320 (49.5%) | Ref. |  |
| satisfaction | 500 (76.2%) | 326 (50.5%) | 0.32 [0.25;0.40] |  |
| Marriage Satisfaction: |  |  |  | <0.001 |
| dissatisfaction | 72 (11.0%) | 171 (26.5%) | Ref. |  |
| satisfaction | 584 (89.0%) | 475 (73.5%) | 0.34 [0.25;0.46] |  |
| Chidren Satisfication: |  |  |  | <0.001 |
| dissatisfaction | 13 (1.98%) | 55 (8.51%) | Ref. |  |
| satisfaction | 643 (98.0%) | 591 (91.5%) | 0.22 [0.11;0.39] |  |
| Air Quality Satisfaction: |  |  |  | 0.004 |
| dissatisfaction | 101 (15.4%) | 141 (21.8%) | Ref. |  |
| satisfaction | 555 (84.6%) | 505 (78.2%) | 0.65 [0.49;0.86] |  |
| IADL: |  |  |  | <0.001 |
| Difficulties | 91 (13.9%) | 192 (29.7%) | Ref. |  |
| No Difficulties | 565 (86.1%) | 454 (70.3%) | 0.38 [0.29;0.50] |  |
| Health during Childhood: |  |  |  | 0.391 |
| good | 490 (74.7%) | 468 (72.4%) | Ref. |  |
| not good | 166 (25.3%) | 178 (27.6%) | 1.12 [0.88;1.44] |  |
| Troubled with Body Pain: |  |  |  | <0.001 |
| no | 198 (30.2%) | 105 (16.3%) | Ref. |  |
| yes | 458 (69.8%) | 541 (83.7%) | 2.22 [1.71;2.91] |  |
